# Cleavage of functional IL-2 receptor alpha chain (CD25) from murine corneal and conjunctival epithelia by MMP-9

**DOI:** 10.1186/1476-9255-6-31

**Published:** 2009-10-31

**Authors:** Cintia S De Paiva, Kyung-Chul Yoon, Solherny B Pangelinan, Sapa Pham, Larry M Puthenparambil, Eliseu Y Chuang, William J Farley, Michael E Stern, De-Quan Li, Stephen C Pflugfelder

**Affiliations:** 1 Ocular Surface Center, Department of Ophthalmology, Cullen Eye Institute, Baylor College of Medicine, Houston, Texas, USA; 2 Department of Ophthalmology, Chonnam National University, Medical School and Hospital, Gwangju, South Korea; 3 Allergan, Irvine, CA, USA

## Abstract

**Background:**

IL-2 has classically been considered a cytokine that regulates T cell proliferation and differentiation, signaling through its heterotrimeric receptor (IL-2R) consisting of α (CD25), β (CD122), γ chains (CD132). Expression of IL-2R has also been detected in mucosal epithelial cells. Soluble IL-2Rα (CD25) has been reported as an inflammatory marker. We evaluated the expression of CD25 and CD122 in the ocular surface epithelium and investigated the mechanism of proteolytic cleavage of CD25 from these cells.

**Methods:**

Desiccating stress (DS) was used as an inducer of matrix metalloproteinase 9 (MMP-9). DS was created by subjecting C57BL/6 and MMP-9 knockout (BKO) mice and their wild-type littermates (WT) mice to a low humidity and drafty environment for 5 days (DS5). A separate group of C57BL/6 mice was subjected to DS5 and treatment with topical 0.025% doxycycline, a MMP inhibitor, administered QID. The expression of CD25 and CD122 was evaluated in cryosections by dual-label laser scanning confocal microscopy. Western blot was used to measure relative levels of CD25 in epithelial lysates. Gelatinase activity was evaluated by in situ zymography. Soluble CD25 in tear fluid was measured by an immunobead assay.

**Results:**

CD25 and CD122 were abundantly expressed in cornea (all layers) and conjunctiva epithelia (apical and subapical layers) in nonstressed control mice. After desiccating stress, we found that immunoreactivity to CD25, but not CD122, decreased by the ocular surface epithelia and concentration of soluble CD25 in tears increased as MMP-9 staining increased. CD25 was preserved in C57BL/6 mice topically treated with an MMP-9 inhibitor and in MMP-9 knock-out mice. MMP-9 treatment of human cultured corneal epithelial cells decreased levels of CD25 protein in a concentration dependent fashion.

**Conclusion:**

Our results indicate that functional IL-2R is produced by the ocular surface epithelia and that CD25 is proteolytic cleaved to its soluble form by MMP-9, which increases in desiccating stress. These findings provide new insight into IL-2 signaling in mucosal epithelia.

## Background

IL-2 is a pleiotropic cytokine that has been identified to play a pivotal role in regulating the adaptive immune response [1]. Its multiple functions include stimulating proliferation of activated T cells (CD4-, CD8-, CD4-CD8+, CD4+ and CD8+ lineage), proliferation and immunoglobulin synthesis by activated B cells, generation, proliferation and activation of NK cells, differentiation and maintenance of FoxP3+CD4+CD25+ T regulatory cells, and activation-induced cell death by increasing the transcription and expression of Fas-Ligand (Fas-L) on CD4+T cells [2-5].

IL-2 signals through its heterotrimeric receptor consisting of α (IL-2Rα, CD25), β (IL-2Rβ, CD122) and γ (IL-2Rγ, CD 132) chains [1,6]. The γ chain, also referred to as the common cytokine receptor chain, is shared by receptors for multiple cytokines including IL-2, IL-4, IL-7, IL-9, IL-15 and IL-21 [7]. IL-2R expression has been detected on non-hematopoetic cells, including mucosal epithelia. The IL-2Rβ chain (CD122) was previously detected on the IEC rat intestinal epithelial cell line and primary rat intestinal epithelial cultures [8]. IL-2 treatment of these intestinal epithelial cells was noted to stimulate production of TGF-β [9].

IL-2Rα is an essential component of the IL-2R. IL-2Rα knock-out mice are phenotypically similar to IL-2 knock-outs, both are resistant to activation-induced cell death and develop severe autoimmunity and lymproliferative syndromes including Sjögren's syndrome (SS) like disease [10-12]. CD25 immunoreactivity in epithelial cells and lymphocytes was previously found in minor salivary glands obtained from patients with SS [13-15]. CD25 expression by the mouse corneal epithelium has also been reported [16].

Soluble CD25, generated by proteolytic cleavage from cells [17,18], is recognized as a marker of inflammation in bodily fluids, including serum, urine and tears [18-21]. Increased levels of CD25 in the serum is considered a marker of disease activity in many systemic autoimmune diseases [22-25], including SS [26,27]. The mechanism by which soluble CD25 is generated in mucosal sites has not been completely elucidated.

We hypothesized that a functional IL-2R is expressed by the ocular surface epithelia and that cell membrane CD25 decreases in dry eye, a condition associated with increased protease activity on the ocular surface.

The purpose of this study was to evaluate if functional IL-2Rα (CD25) is expressed by the ocular surface epithelia (mouse and human) and to evaluate the effects of experimentally induced desiccating stress in mice on cell associated and soluble CD25 in the tears.

## Methods

This research protocol was approved by the Baylor College of Medicine Center for Comparative Medicine and it conformed to the standards in the Association for Research in Vision and Ophthalmology (ARVO) Statement for the use of animals in ophthalmic and vision research.

### Animals and mouse model of dry eye

To evaluate the role of MMP-9 in CD25 expression, we used our murine desiccating stress models (DS) which has been reported to increase MMP-9 activity on the ocular surface [28,29]. DS was induced in 6-8 week old C57BL/6, Jackson Laboratories, Bar Harbor, ME) for 5 days (DS5), without (n = 40) or with (n = 18) topical therapy 4 times a day (1 μL/eye bilaterally of 0.025% doxycycline preservative free, DS5+Doxy, Leiter's Pharmacy, San Jose, CA) as previously reported [28-32]. The doxycycline was freshly prepared and shipped within 24 hours. Doxycycline has been shown to be a MMP inhibitor in a variety of tissues [29,33,34]. A group of age and gender matched C57BL/6 mice (n = 40) without dry eye served as nonstressed controls (NS).

To confirm the role of MMP-9 (gelatinase B) on CD25 expression, DS5 was also induced in MMP-9 knockout mice (referred to as BKO mice, created on a 129SvEv/CD-1 mixed background as previously reported [35], n = 6) and their wild-type littermates of both genders (GelB +/+, referred as WT, n = 6). A separate group of age and gender matched BKO and WT mice (n = 6/strain) without dry eye served as NS controls.

Nonstressed CD25 knock-out (CD25KO, B6.129S4-IL2ra^tm1Dw^/J strain, n = 3) mice were purchased from Jackson Laboratories and were used at 8 weeks of age.

### Exogenous administration of IL-2

To evaluate the role of IL-2 stimulation on Fas-L expression, NS C57BL/6 mice (n = 3) received bilateral subconjunctival injections of recombinant murine IL-2 (10 ng/mL/eye/injection, dissolved in 20 μL of 0.1% bovine serum albumin (BSA) in PBS, R&D Systems, Minneapolis, MN) at days 0, 2 and 4. Vehicle control mice (n = 3) received bilateral subconjunctival injections (20 μL/eye) of 0.1% BSA in PBS on the same schedule. Mice were euthanized on day 5.

### Tear fluid collection and CD25 Luminex Immunobead assay

Tear fluid washings were collected from twelve C57BL/6 mice per group (NS, DS5, DS5+Doxy), and twelve BKO and twelve NLM per group (NS, DS5) in 3 independent experiments using a previously reported method [36]. Briefly, 1.5 μL of PBS+0.1% BSA was instilled into the conjunctival sac. The tear fluid and buffer were collected with a 1-μL volume glass capillary tube (Drummond Scientific Co., Broomhall, PA) by capillary action from the tear meniscus in the lateral canthus) and stored at -80°C until the assay was performed. One sample consisted of tear washings from both eyes of two mice pooled (4 μL) in mouse CD25 assay buffer (6 μL). There were a total of 6 samples from each group of mice.

CD25 concentrations in tear washings of NS, DS5 and DS5+Doxy groups were measured using a sensitive, fluorescent bead-based sandwich immuno assay (Biosource, Invitrogen, Carlsbad, CA). Briefly, 10 μL of murine tear washings or buffer alone (blank controls) were added to wells containing the appropriate 1× beads coupled to anti-CD25 antibody. Serial dilutions of CD25 were added to wells in the same plate as the tear samples to generate a standard curve. The plate was incubated overnight at 4°C to capture CD25 by the antibody-conjugated fluorescent beads. After 3 washes with assay buffer, 100 μl of 1× biotinylated specific reporter antibody for CD25 mixture was applied for 1 hour in the dark at room temperature. The reaction was detected with streptavidin-phycoerythrin with a Luminex 100 IS 2.3 system (Austin, TX). The results are presented as pg/mL. The experiments were repeated in 3 different sets of animals and the results were averaged.

### Laser scanning confocal immunofluorescent microscopy

Expression of IL-2Rα and IL-2Rβ chains (CD25 and CD122, respectively), MMP-9 and Fas-L was evaluated by laser scanning confocal microscopy in cryosections of murine eyes, human cornea and conjunctiva.

The right eyes and lids of mice from each group were excised (n = 6 right eyes/group), embedded in OCT™ compound (VWR, Swannee, GA), and flash frozen in liquid nitrogen. Sagittal 8-μm sections were cut with a Micron HM 500 cryostat (Waldorf, Germany) and stored at -80°C.

Fresh human corneoscleral tissues and conjunctiva (preserved in less than 8 hour postmortem) that were not suitable for clinical use (donors aged 19-64 years, n = 4), were obtained from the Lions Eye Bank of Texas (Houston) or from the National Disease Research Interchange (Philadelphia). They were cut through the horizontal meridian, frozen, and sectioned as described above.

Cryosections stained for CD25 (clone 7D4, 5 μg/mL, BD Pharmingen, San Jose, CA), MMP-9 (10 μg/mL, Chemicon-Millipore, Billerica, MA), CD122 or Fas-L (rabbit polyclonal antibodies, 5 μg/mL and 4 μg/mL, respectively, both from Santa Cruz Biotechnology, Santa Cruz, CA) were developed using appropriated Alexa-Fluor 488 conjugated IgG antibodies as previously described [32,37]. Negative controls were performed at the same time and consisted of sections incubated with an isotype control antibody or sections with omitted primary antibody. Nuclei were counterstained with propidium iodide (2 μg/ml in PBS) to yield a red color.

Dual label for CD25 and CD122 was performed by simultaneous incubation of both antibodies, followed by extensive washing and simultaneous incubation of both secondary antibodies (Alexa-Fluor 488 conjugated goat anti-rat IgG and Alexa-Fluor 633 conjugated goat anti-rabbit IgG, 1:300 dilution) in a dark chamber. The co-localization of CD25 labeled in green and CD122 labeled in blue yielded a turquoise color in merged images.

Digital images (512 × 512 pixels) were captured with a laser-scanning confocal microscope (LSM 510, Zeiss with krypton-argon and He-Ne laser; Zeiss, Thornwood, NY) with either 488 excitation and 543 nm excitation emission filters (LP505 and LP560, for single labeling) or 488, 543 nm and 633 nm excitation emission filters (BP505-550, BP 560-615 and LP 650, for dual labeling). They were acquired with a 40/1.3× oil-immersion objective. Images from DS and NS groups were captured with identical photomultiplier tube gain settings and processed using Zeiss LSM-PC software and Adobe Photoshop 6.0 (Adobe Inc., San Jose).

### Measurement of fluorescence intensity in cornea

Fluorescence intensity of CD25, CD122, MMP-9 and Fas-L in digital images of cornea and conjunctiva captured by laser scanning confocal immunofluorescent microscopy was measured using NIS Elements Software (version 3.0, BR, Nikon, Melville, NY). At least 6 images/time point/strain were analyzed. The epithelial layer of the stained cornea/conjunctiva was circumscribed by 2 masked observers and the mean fluorescence intensity was calculated by the software and entered into Excel (Microsoft Corp, Redmond, WA) and the results average within each group (Microsoft Corp, Redmond, WA). Data is presented as mean ± standard deviation of gray levels.

### In situ zymography

In situ zymography was performed to localize the gelatinase activity in corneal cryosections obtained from C57BL/6, BKO and WT mice (n = 6 per strain/time point) as previously reported [29]. Sections were thawed and incubated overnight with reaction buffer, 0.05 M Tris-HCl, 0.15 M NaCl, 5 mM CaCl_2_, and 0.2 mM NaN_3_, pH 7.6, containing 40 μg/mL FITC-labeled DQ gelatin (EnzChek, Molecular Probes, Eugene, OR). As a negative control, 50 μM of 1, 10-phenanthroline, a metalloproteinase inhibitor, was added to the reaction buffer before applying the FITC-labeled DQ gelatin to frozen sections. After incubation, the sections were washed three times with PBS for 5 minutes and counterstained with propidium iodide (2 μg/ml in PBS) for 5 minutes and were covered with anti-fade Gel/Mount (Fisher, Atlanta, GA) and coverslips. Areas of gelatinolytic activity were imaged by a Nikon DXM 1200 digital camera (Nikon, Garden City, NY). Proteolysis of the FITC-labeled DQ gelatin substrate at sites of net gelatinase activity yields fluorescent gelatin-FITC peptides and the intensity is proportional to the amount of activity within in the tissue.

### Corneal epithelial explant cultures

Human corneal epithelial cells were cultured from explants taken from human corneoscleral rims, provided by the Lions Eye Bank of Texas, using a previously described method [38,39]. Corneal explants were grown in a 6 well plate or on an eight-chamber slide (Nunc Lab-Tek II, Nalge Nunc International Corp, Naperville, IL).

### MMP-9 treatment of cultured human corneal epithelium

Except for the control group that were maintained in serum-free culture media, the confluent corneal epithelial cultures were exposed to increasing concentrations of MMP-9 (100, 250 and 500 ng/mL, Calbiochem, EMD Chemicals, Inc., San Diego, CA) for 48 hours. After 48 hours, the adherent cells were exposed to lysis buffer B (Upstate, Lake Placid, NY) and an EDTA-free protease inhibitor cocktail tablet (Roche Applied Science, Indianapolis, IN), for Western blot analysis. Three experiments were performed using separate sets of cultures that were initiated from different donor corneas. Cells were grown in either a 6 well culture plate (which were used for Western blot analysis) or an eight chamber slides (which were processed for CD25 immunostaining as described above).

### Western blot

Scraped mouse corneal epithelia and surgically excised conjunctiva, collected from NS and DS5 C57BL/6 mice (n = 4 animals/time point/3 independent sets of experiments), were separately pooled and lysed in a buffer containing 1% Triton X-100, 100 mM NaCl, 10 mM HEPES, 2 mM ethylenediaminetetraacetic acid (EDTA) and an EDTA-free protease inhibitor cocktail tablet and centrifuged at 15,000 × *g *for 30 minutes at 4°C. The total protein concentrations of the cell extracts were measured by a Micro BCA protein assay kit (Pierce, Rockford, IL).

Protein samples (75 μg/lane) were separated by SDS-polyacrylamide gel electrophoresis (4 to 15% Tris-HCl, gradient gels; Bio-Rad, Hercules, CA), and transferred electronically to polyvinylidene difluoride membranes (Millipore, Bedford, MA). The membranes were blocked with 5% nonfat milk in TTBS (50 mM Tris, pH 7.5, 0.9% NaCl, and 0.1% Tween-20) for 1 hour at room temperature, and then incubated overnight at 4°C with a monoclonal rat antibody anti-CD25 (clone 7D4, 10 μg/mL, BD Pharmingen, San Jose, CA) with 5% nonfat milk in TTBS. After washing with TTBS, the membranes were incubated for 1 hour at room temperature with HRP-conjugated secondary antibody goat anti-rat IgG (1:2000 dilution; Pierce, Rockford, IL). The signals were detected using the ECL plus Western Blotting Detection System (Amersham Biosciences, Little Chalfort Buckinghamshire, England) and the images were acquired and analyzed by a Kodak Image Station 2000R (Eastman-Kodak, New Haven, CT). Bands intensities were measured with Kodak 1D v3.6 software. The data is presented as the mean ± error mean of 3 independent experiments using arbitrary units.

## Results

### Desiccating stress induces gelatinolytic cleavage of CD25 from the ocular surface epithelia

**Figure 1:**
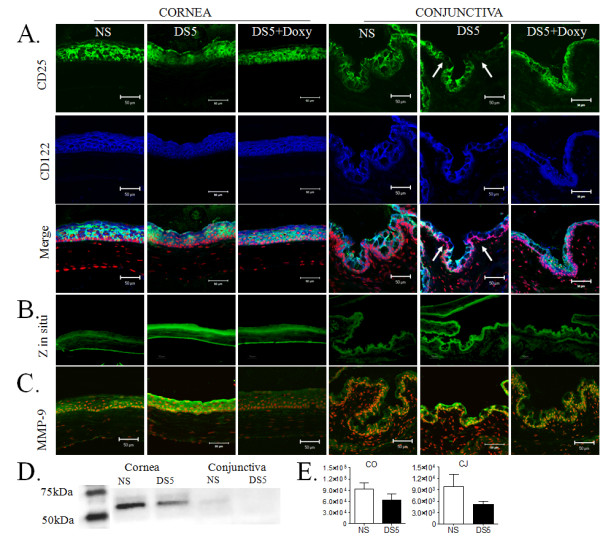
**A: Dual label immunofluorescent laser scanning confocal microscopy of ocular surface tissue sections from C57BL/6 mice for interleukin 2 receptor alpha (CD25, green) and beta chains (CD122, blue) with propidium iodide (red) nuclear counterstaining in nonstressed controls (NS), 5 days (D) of desiccating stress (DS5) and DS5 treated with topical doxycycline (DS5+Doxy) in C57BL/6 mice**. A turquoise color indicates co-localization of both markers. Note partial disappearance of CD25 with preservation of CD122 after DS5 (arrows) in the conjunctival epithelia. Scale bar = 50 μm **1. B**. Tissue sections prepared for in situ zymography (in situ Z) in nonstressed controls (NS), 5 days (D) of desiccating stress (DS5) and DS5 treated with topical doxycycline (DS5+Doxy) in C57BL/6 mice. Scale bar = 100 μm. **1. C**. Merged images of laser scanning confocal fluorescent microscopy of ocular surface tissue sections stained for matrix metalloproteinase 9 (MMP-9, in green) with propidium iodide (PI, red) nuclear counterstaining in NS controls, DS5 and DS5+Doxy groups in C57BL/6 mice. Scale bar = 50 μm **1. D**. Representative Western blot showing effect of DS on CD25 expression in corneal (CO) and conjunctival epithelial (CJ) lysates. **1. E**. Bar graphs are mean + standard error mean of CD25 band intensities in 3 independent Western blots (arbitrary units).

**Figure 2:**
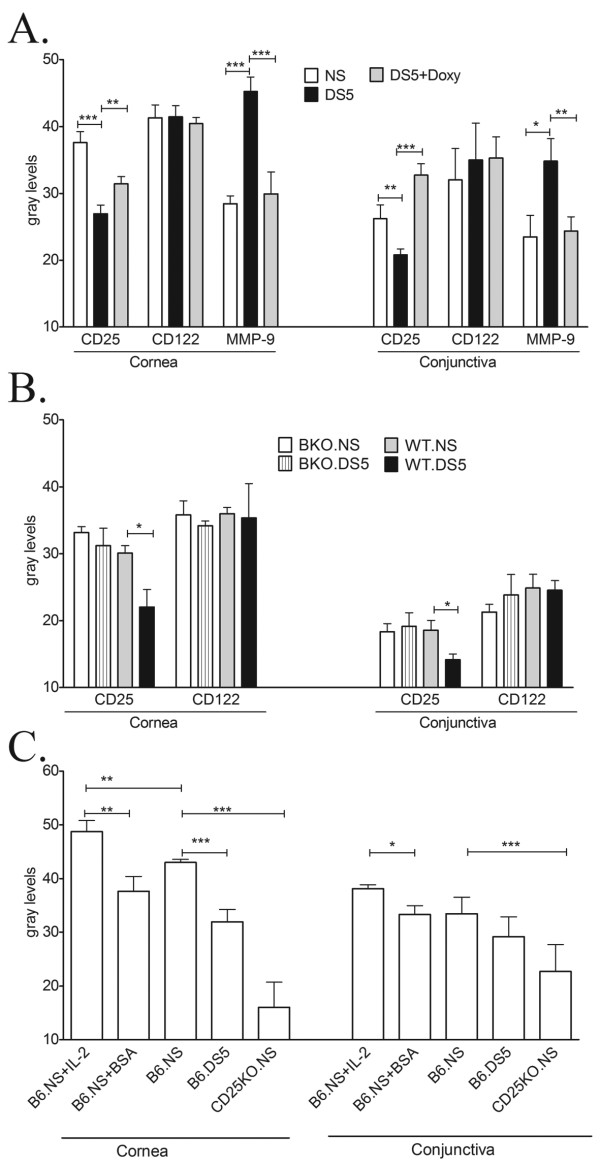
**Mean ± standard deviation of fluorescent intensity measurements in corneal and Conjunctival epithelia stained for CD25, CD122, MMP-9 in nonstressed (NS) C57BL/6 (A) or BKO and WT (B) and mice subjected for desiccating stress for 5 days (DS5)**. A separate group of DS5 mice were topically treated with doxycycline (DS5+Doxy) (C) Mean ± standard deviation of fluorescent intensity measurements in corneal and conjunctival epithelia stained for Fas-L in nonstressed (NS) C57BL/6 mice (B6-NS), desiccating stressed C57BL/6 mice for 5 days (B6-DS5), NS C57BL/6 mice treated with bovine serum albumin (injection control, B6-NS+BSA) or IL-2 subconjunctival injections (B6-NS+IL-2), CD25 knock-out mice (CD25KO-NS). *P < 0.05; ** = P < 0.01; *** = P < 0.001.

The presence and localization of CD25 and CD122 in the ocular surface epithelia were investigated by immunofluorescent staining (Figure 1) and the intensity of the staining was analyzed in digital images (Figure 2A). Using dual label laser scanning immunofluorescent microscopy, both IL-2R chains were present in all layers of the corneal epithelium and in the apical and subapical layers of the conjunctival epithelium of C57BL/6 mice (Figure 1A). The level of expression of both IL-2R chains was higher in the corneal than in the conjunctival epithelia (Figure 2A).

Desiccating stress caused a marked significant loss of CD25 in all corneal and conjunctival epithelial layers, while the CD122 staining intensity remained unchanged (Figure 1A, 2A). In many areas in the conjunctiva, loss of CD25 with no change in CD122 expression was observed (Figure 1A, arrows). To confirm the loss of CD25, Western blot was performed in corneal and conjunctival epithelial lysates from NS controls and DS5 C57BL/6 mice, in 3 independent experiments. As shown in Figure 1(D-E), there is a clear decrease in the intensity of the CD25 band in the DS5 in corneal and conjunctival epithelia.

To investigate the role of gelatinases (MMP-2, MMP-9) in the loss of CD25 in response to desiccating stress, in situ zymography was performed in 3 different samples obtained from NS and DS5 CD57BL/6 mice (Figure 1B). Compared to control eyes, higher gelatinolytic activity was noted in both the corneal and the conjunctival epithelia after DS5. To determine if gelatinase activity was due to an increase in MMP-9 expression, we performed immunostaining for MMP-9 in sequential slides. Desiccating stress was also noted to significantly increase immunoreactivity to MMP-9 in both cornea and conjunctiva epithelia, compared to nonstressed controls (Figure 1C, 2A).

To investigate the role of MMP-9 in the loss of CD25 from ocular surface epithelia, a separate group of mice were treated with the MMP inhibitor, doxycycline. Topically applied doxycycline prevented CD25 loss, while decreasing DS-induced gelatinase activity and MMP-9 staining in corneal and conjunctival epithelium (Figure 1A-C; 2A).

### Soluble CD25 is present in tear fluid of C57BL/6 mice

To determine if CD25 is shed from the ocular surface epithelia into the tears, the presence of soluble CD25 was evaluated in tear fluid washings obtained from NS and DS5 mice. Soluble CD25 was higher in tears of DS5 than NS mice (59.40 ± 1.13 vs. 27.22 ± 26.48 ng/mL, respectively, P < 0.05). Compared to the levels in the DS5 group, topical treatment with the metalloproteinase inhibitor doxycycline (DS5+Doxy) decreased the levels of soluble CD25 in tears (9.87 ± 3.67 ng/mL, P < 0.01).

### MMP-9 knock-out confers resistance to CD25 loss

**Figure 3:**
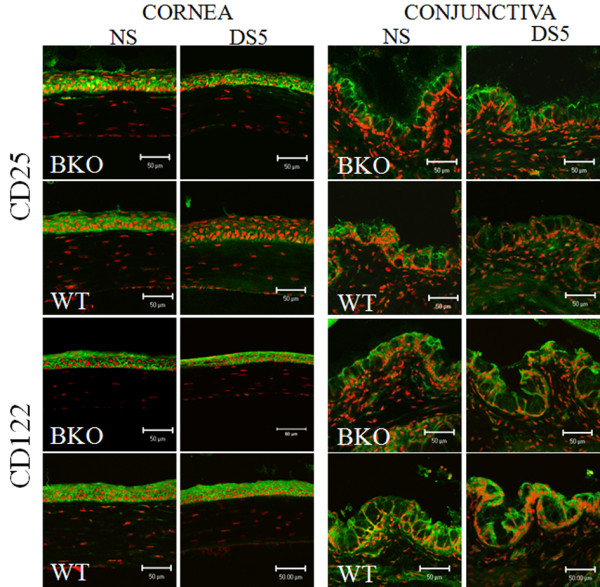
**Merged images of laser scanning confocal fluorescent microscopy ocular surface tissue sections stained for interleukin 2 receptor alpha chain (CD25, in green) and interleukin 2 receptor beta chain (CD12, in green) with propidium iodide (PI, red) nuclear counterstaining in nonstressed controls (NS), 5 days (D) of desiccating stress (DS5) in MMP-9 knock-out (BKO) and wild-type (WT) mice**. All images shown are the merged image of CD25 and CD122 (in green) with PI counterstaining. Scale bar = 50 μm.

To confirm the specific role of the gelatinase MMP-9 in the loss of CD25 from the ocular surface epithelia in response to desiccating stress, experimental dry eye was induced in BKO and their WT littermates for 5 days. Immunostaining for CD25 and CD122 was performed in cryosections (Figure 3) and intensity of immunoreactivity was measured in the epithelial layer (Figure 2B).

CD25 and CD122 immunoreactivity were found in all layers of corneal epithelia and suprabasal and apical layers of conjunctiva in NS BKO and WT (Figure 3), in a similar pattern to NS C57BL/6 mice. However; the staining intensity was weaker than seen in C57BL/6, perhaps be due to different genetic backgrounds between the two strains. DS5 caused a significant decrease in CD25 expression in both corneal and conjunctival epithelia in WT mice, while CD25 immunoreactivity was preserved in the BKO mice, with no concomitant change in CD122 immunoreactivity in either strain (Figure 2B and 3).

We also measure the presence of soluble CD25 in tear fluid washings obtained from BKO and WT before and after desiccating stress for 5 days. Soluble CD25 was higher in tears of DS5 WT mice than NS WT (10.1 ± 5.66 vs. 4.8 ± 4.2 ng/mL, respectively, P < 0.05). No change in the levels of soluble CD25 in tears of BKO mice were observed between NS and DS5 (6.05 ± 5.64 vs. 5.01 ± 4.5 ng/mL, P > 0.05).

### CD25 appears to be a functional IL-2 receptor

One of the functions of IL-2 is to stimulate production of Fas-L[5]. Fas-L has been previously found to be expressed by the corneal epithelium and endothelium [40] where it is considered to play an important role in the establishment and maintenance of immune privilege by inducing apoptosis of lymphocytes. To determine if CD25 is a component of a functional IL-2R on the ocular surface epithelium, we evaluated Fas-L expression in eyes with normal and reduced levels of CD25 and after exogenous administration of IL-2 for 5 days.

**Figure 4:**
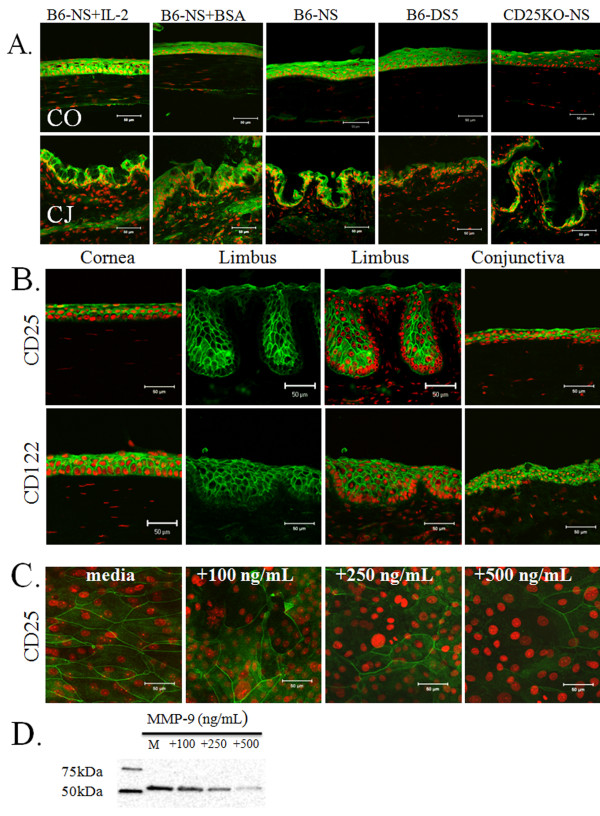
**Merged images of laser scanning confocal fluorescent microscopy of cornea (CO) and conjunctiva (CJ) sections stained for Fas-ligand (green) with propidium iodide (PI, red) nuclear counterstaining in nonstressed (NS) C57BL/6 mice (B6-NS), desiccating stressed C57BL/6 mice for 5 days (B6-DS5), NS C57BL/6 mice treated with bovine serum albumin (injection control, B6-NS+BSA) or IL-2 subconjunctival injections (B6-NS+IL-2), CD25 knock-out mice (CD25KO-NS)**. Scale bar = 100 μm **3. B**. Laser scanning confocal fluorescent microscopy of human tissue sections (cornea, limbus and conjunctiva) stained for interleukin 2 receptor alpha chain (CD25, green) with PI (red) nuclear counterstaining. For the limbus, the CD25 and CD122 image (in green) is shown besides the merged image with PI nuclear staining. Note absence of staining on the basal epithelial layer of the limbus. Scale bar = 50 μm **3. C**. Laser scanning confocal microscopy of human corneal epithelial cells grown on an eight chamber slide and stained for CD25 after treatment with increasing concentrations of MMP-9 for 48 hours. Scale bar = 50 μm **3. D**. Representative Western blot showing effect of MMP-9 treatment on CD25 expression by cultured human corneal epithelial cells lysates. Increasing MMP-9 concentration decreased CD25 levels.

Fas-L expression was evaluated by immunofluorescent scanning confocal microscopy in NS eyes after subconjunctival injection of IL-2 or vehicle alone (BSA) (Figure 4), in 3 different C57BL/6 mice and intensity of immunoreactivity was measured in the epithelial layer (Figure 2C). Significant increased Fas-L immunostaining was observed in all layers of the conjunctival and corneal epithelia in IL-2 injected eyes compared to vehicle injected eyes (Figures 2C and 4A). Both DS5 C57BL/6 and NS CD25KO mice exhibited significantly lower levels of immunoreactivity to Fas-L in the corneal epithelia compared to NS C57BL/6 (Figures 2C and 4A). The lowest level of Fas-L immunoreactivity was seen in the NS CD25KO cornea (Figure 2C). Taken together, these findings indicate that CD25 is a component of a functional IL-2 receptor on the ocular surface epithelia.

### MMP-9 cleaves CD25 in cultured human corneal epithelial cells

We initially confirmed the presence of CD25 and CD122 in the human ocular surface epithelia by laser scanning immunofluorescent microscopy in cryosections of central cornea, limbus and conjunctiva (Figure 4B) and in human cultured corneal epithelial cells. CD25 and CD122 immunoreactivity was found in all layers of human central cornea and conjunctiva, but it was absent in the basal layer of limbus (Figure 4B). In primary human cultured corneal epithelial cells, we observed that CD25 immunoreactivity was strong in the larger superficial differentiated cells, in stratified cultures, while weaker staining was noted in basal cells of (data not shown).

To determine if CD25 could be cleaved from the ocular surface epithelia cells by MMP-9, we exposed human cultured corneal epithelia to recombinant MMP-9. Treatment of these cultured cells with increasing concentrations of MMP-9 progressively decreased membrane CD25 immunoreactivity, with very minimal staining observed following exposure to MMP-9 500 ng/mL (Figure 4C). Western blot revealed that MMP-9 treatment decreased cell associated CD25 levels in a concentration-dependent manner (Figure 4D).

## Discussion

Our studies found that the unique chains of the IL-2R (CD25 and CD 122) are expressed by the ocular surface epithelia in mice and humans. In humans, CD25 and CD122 were produced by all cell layers in central cornea and conjunctiva, while they were expressed by the apical epithelium of the corneal limbus in a differentiation dependent fashion. The lack of IL-2R receptors in the limbal basal layer, the site of putative corneal epithelial stem cells, deserves further investigation.

Desiccating stress in mice was found to decrease CD25 immunoreactivity in the corneal and conjunctival epithelium and increase soluble CD25 in tears. This decrease appeared to be due in part to proteolytic cleavage by MMP-9 because there was no change in the level of CD25 expression in MMP-9 deficient mice and after topical treatment of mice exposed to DS with the metalloproteinase inhibitor doxycycline. Furthermore, treatment of primary human cultured corneal epithelium with MMP-9 induced a dose-dependent loss of CD25 from the cell surface.

We have previously found that dry eye and desiccating stress stimulate production of MMP-9, as well as other MMPs by the ocular surface epithelia [28,29,41], while no changes in the tissue inhibitor of MMPs was observed[31] MMP-9 was found to degrade the tight-junction protein occludin and to disrupt apical epithelial barrier function in the cornea[28,29] Furthermore, MMP-9 knock-out mice were found to be resistant to the corneal epithelial disease that develops in response to dry eye[28] MMP-9 in human tears has also been found to increase in a variety of ocular surface diseases, including sterile corneal ulceration [42-46] In a group of dry eye patients, we observed that tear MMP-9 activity levels increased as the severity of corneal disease progressed. Tear MMP-9 activity levels also correlated positively with corneal fluorescein staining scores and with low contrast visual acuity in this study[47]

It is worth noting that the role of MMP-9 in the cleavage of soluble CD25 is still controversial. High doses of MMP-9 (1 ug/mL) were shown to downregulate the expression of IL-2Rα on activated human T cells [18]. Another study demonstrated that treatment of Kit225 leukaemic cells with recombinant MMP-9 slightly decreased membrane CD25 expression and increased the concentration of sIL-2Rα in the supernatants. [48] However, a selective inhibitor of MMP-9 failed to inhibit the release of sIL-2Ra by the Kit225 cell line or by phytohaemagglutinin (PHA)-activated peripheral blood mononuclear cells, while a broad MMP-inhibitor such as TAPI-0 succeeded. [48]

Using MMP-9 knock-out mice on a C57BL/6 background, El Houda Agueznay and colleagues did not observe differences in baseline serum soluble CD25 concentrations and in soluble CD25 production by activated T cells compared to wild-type mice [48]. Our in vitro studies support a role for MMP-9 in cleaving cell membrane CD25. We found a decrease in cell associated CD25 when human corneal epithelial cells were treated with high concentrations of MMP-9 (Figure 4). Furthermore, our in vivo results demonstrated a significant increase in soluble CD25 in tear fluid of WT subjected to desiccating stress, compared to non-stressed mice, whereas there was bi measureable change in concentration of soluble CD25 in tears of BKO mice subjected to similar environmental conditions.

CD25 on the ocular surface epithelium appears to be a component of a functional IL-2R. We found that exogenous IL-2 stimulation increased expression of Fas-L by the surface epithelia, while mice subjected to DS and those lacking the alpha portion of IL-2 receptor had low levels of Fas-L.

The concentration of soluble CD25 in tears may prove to be a valuable indicator of the level of proteolytic activity on the ocular surface epithelium. Significantly increased tear concentrations of CD25 have previously been noted in a number of ocular surface diseases, including vernal and atopic keratoconjunctivitis, seasonal allergic conjunctivitis and rosacea blepharoconjunctivitis [21]. Increased tear protease activity on the ocular surface has been reported in many of these conditions [8,9,16].

IL-2 has previously been detected in tear fluid [36,49,50] and based on the findings of our study it is possible that IL-2 may play a vital role in maintaining homeostasis on the ocular surface. IL-2 has been found to stimulate secretion of the key immunoregulatory cytokine TGF-β by cultured intestinal epithelial cells and it may have a similar role on the ocular surface [9]. These studies prove the rationale for further investigation of the role of IL-2 signaling on the ocular surface.

## Abbreviations used in this manuscript

Fas-L: Fas-ligand; IL-2Rβ: interleukin 2 receptor beta chain; IL-2Rα: interleukin 2 receptor alpha chain; sIL-2Rα: soluble interleukin 2 receptor alpha chain; SS: Sjögren's syndrome; MMP-9: matrix metalloproteinase 9; BKO: gelatinase B (MMP-9) knock-out mice strain; WT: wild-type; CD25KO: CD25 knock-out mice strain; DS: desiccating stress; DS5: desiccating stress for 5 days; NS: nonstressed; PI: propidium iodide.

## Competing interests

ME Stern is an employee of Allergan, Irvine, CA. The other authors have no competing interests.

## Authors' contributions

The manuscript was written and experiments designed by CSDP and SCP. All experiments were performed by CSDP, KCY, SBP, SP, LP, EYC and WJF and supervised by SCP and DQL, who also oversaw manuscript construction together with MES. All authors have given final approval of the version to be published.
